# Bone Health in Handball Players: The Role of Exercise

**DOI:** 10.7759/cureus.95883

**Published:** 2025-11-01

**Authors:** Zacharoula Papadopoulou, Eleni Maria Vrampa, Angelo V Vasiliadis, Nikiforos Galanis

**Affiliations:** 1 1st Orthopedic Department, George Papanikolaou General Hospital, Aristotle University of Thessaloniki, Thessaloniki, GRC; 2 Competitive Sports Department, School of Physical Education and Sports Science, Aristotle University of Thessaloniki, Thessaloniki, GRC; 3 Sports Trauma and Orthopedic Department, St. Luke’s Hospital, Thessaloniki, GRC; 4 School of Medicine, Aristotle University of Thessaloniki, Thessaloniki, GRC

**Keywords:** bone health, bone mineral content, bone mineral density, exercise training, handball

## Abstract

Exercise has an indirect but significant effect on bone mass by generating mechanical stress on the skeleton. It is widely recommended to enhance bone quality and counteract the loss of bone and muscle mass, particularly in the elderly. Dual-energy X-ray absorptiometry (DEXA) is a relatively inexpensive and effective method for assessing bone mineral density (BMD) and bone mineral content (BMC), both of which are strong predictors of fracture risk. Team handball is a high-impact sport, which is characterized by frequent jumping, landing, and torsional strains on the upper and lower limbs, as well as the spine. These mechanical loads are important regulators of bone mass. The literature highlights the potential positive role of handball training and participation on both BMD and BMC. Specifically, handball players have been shown to exhibit higher BMD and BMC values compared to age-matched active and inactive individuals. The observed improvements in bone parameters among handball players may be attributed to repetitive muscle contractions and the high mechanical loading associated with the sport, which stimulate bone tissue activation and remodeling. Furthermore, a significant difference has been observed between dominant and non-dominant limbs in handball players, with the dominant side showing higher BMD and BMC values in both upper and lower extremities. This review underscores the beneficial role of high-impact sports, such as handball, in promoting bone health, preventing bone loss, and reducing the risk of osteopenia and osteoporosis later in life.

## Introduction and background

The health of the skeletal system is important for athletes of all ages, and it is a critical factor in the prevention of osteopenia and osteoporosis [1]. Bone health can be influenced by both non-modifiable and modifiable factors. Non-modifiable factors include gender, family history, race, and early menopause, while modifiable factors include poor nutrition, lifestyle behaviors (such as smoking and excessive caffeine and alcohol consumption), a sedentary life, and low physical activity [1,2]. Young adulthood is a key growth phase and represents the most beneficial period for increasing bone mineral density (BMD), with nearly 90% of peak BMD attained between the ages of 16 and 30 in both genders [3,4]. However, an imbalance between physical activity and dietary disorders, the low intake of both vitamin D and calcium, and hypoestrogenism can negatively impact bone health in this population [4].

Physical activity plays a crucial role in maintaining bone homeostasis and improving bone health in both young and older adults while reducing age-related bone mass loss [4]. Maintaining a physically active lifestyle can prevent osteopenia and osteoporosis, regulate bone mass maintenance, and reduce the overall risk of falls and fractures [5]. Exercise is a subset of physical activity that is planned, structured, repetitive, and focused on improving or maintaining physical fitness, bone health, and the quality of life [6]. Previous studies have shown that exercise effectively stimulates skeletal muscle hypertrophy and bone formation across the lifespan [7].

**Figure 1:**
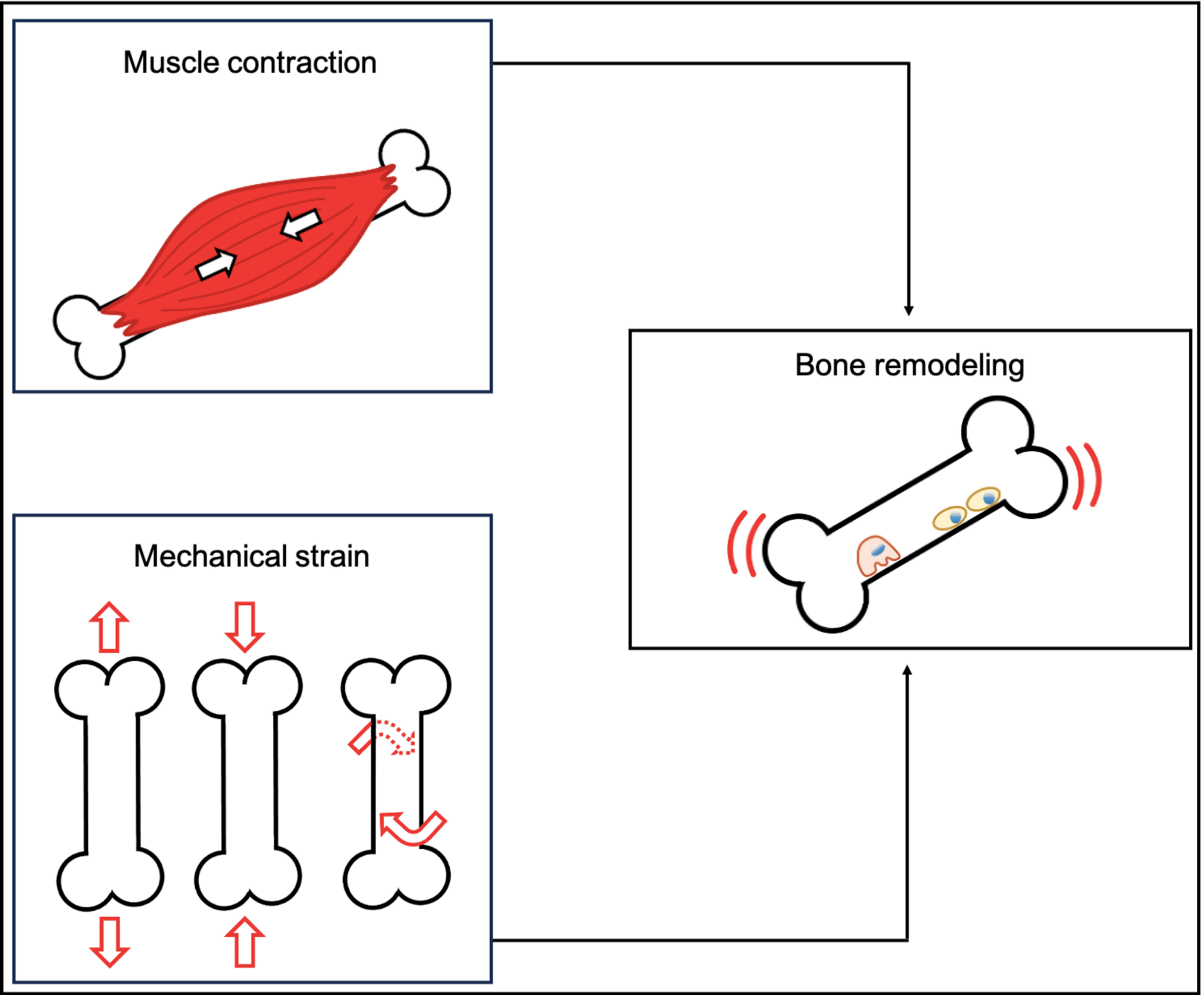
Muscle contraction and mechanical strain, applied at a specific bone, stimulate the activation of bone tissue to the cellular level and promote bone remodeling Figure created by the authors

In general, muscle contraction and mechanical strain are crucial factors in stimulating bone remodeling in living organisms during exercise (Figure 1) [1,7]. The literature supports that those athletes participating in high-impact sports, such as basketball, handball, and jumping sports, have significantly greater BMD compared to those involved in low-impact sports, such as swimming and cycling [1]. Interestingly, the osteogenic effect of exercise appears to be site-specific. Resistance training of the lower limbs combined with jumping exercises seems particularly beneficial for increasing BMD in the lower limb, especially at the neck of the femur and the lumbar spine [8]. Additionally, handball players are examples of athletes who demonstrate greater BMD in the dominant arm, likely an adaptive response to the mechanical stimulus from throwing and bouncing the ball [9].

## Review

Narrative review

Review Methodology

Because our purpose was to summarize and critically discuss the current evidence on bone health and exercise, with a particular focus on handball athletes, our literature review was designed to be comprehensive but not systematic. The initial search was conducted in January 2025 using PubMed and Google Scholar, with the following keywords entered by the lead author: “bone health,” “bone mineral density,” “bone mineral content,” “exercise training,” and/or “handball.” No review articles were identified that specifically addressed bone health in handball players. Additional searches were performed by the authors between January and March 2025 to identify further relevant studies. Secondary articles were located through the reference lists of the primary sources. The included studies focused on active individuals, including handball athletes. As the search yielded a limited number of studies examining bone health in handball players and the effects of exercise, we elected to write a narrative review on this topic.

Evaluating bone mineral density and bone mineral content (BMC)

Bone health is influenced by bone quality (structure and microarchitecture) and bone quantity (bone mineral density). Alterations in bone microarchitecture can significantly affect bone mechanical behavior and may explain the high prevalence of low-impact fractures [10]. Moreover, the loss of bone quantity may weaken the bones and lead to osteopenia and/or osteoporosis, increasing the risk of fractures in the aging population [10]. Dual-energy X-ray absorptiometry (DEXA) is a relatively inexpensive and cost-effective method of BMD measurement [10,11]. In clinical practice, DEXA is a valuable method for the accurate diagnosis of osteoporosis, for the estimation of the risk for fracture, and for monitoring patients treated for osteoporosis [11]. It is well known that aging has a close relationship with changes in BMD, including markedly increased bone resorption over bone formation, especially in postmenopausal women due to the low levels of estrogens. This imbalance is associated with increased disability, morbidity, and mortality [12]. On the contrary, exercise can effectively stimulate bone osteogenesis and improve BMD [12,13]. Regular exercise is key to preventing age-related bone changes and reducing the risk of fractures across the lifespan [13].

While DEXA is the most commonly used method to evaluate BMD, it has limitations, particularly in patients with chronic systemic inflammatory diseases, such as ankylosing spondylitis [14]. High-resolution peripheral quantitative computed tomography (HR-pQCT) is an alternative method for this population, in order to assess the bone microarchitecture with much higher spatial resolution and low radiation dose [14,15]. HR-pQCT can produce precise 3D characterization of the bone and quantify the microstructure, densitometric, and mechanical properties of human cortical and trabecular bone [16]. Alternative imaging modalities for assessing BMD can be the pulsed echo ultrasound, quantitative ultrasound, magnetic resonance imaging, EOS imaging, and digital X-ray radiogrammetry [16,17]. Monitoring bone health is essential in clinical research for evaluating disease progression, treatment response, and bone adaptations due to exercise.

The evaluation of bone mineral content (BMC), the amount of mineral in a specific bone site, can also be a strong predictor of fracture risk. DEXA can measure BMC at both total and regional levels and is highly valued for its reproducibility [18]. In addition, the high sensitivity of the DEXA makes this technique applicable to small bones [19]. BMD and BMC are among the body composition variables that must be included in studies performed according to the International Society for Clinical Densitometry [18]. BMC increases with age from birth through childhood [20], showing the strongest association with height gain in early childhood and a positive correlation with birth weight and body mass index at birth [21].

Effects of exercise on the bone

Exercise has beneficial effects on bone health and appears to play a protective role in preventing osteoporosis and reducing fracture risk [1,5,13]. However, not all types of exercises have the same impact on the bones [22]. For example, walking provides a modest increase in the loads of the bone skeleton, proving to be less effective in the prevention of osteoporosis [22]. Interestingly, unloaded exercises, such as swimming and cycling, have no impact on the bone mass of the lower extremities [23]. In contrast, weight-bearing activities, such as long-distance running and resistance training, positively affect bone development and increase BMD, especially at the hip and spine [8,24].

Sports that involve high muscle tension in upper extremities, such as tennis and handball, promote positive bone adaptations by enhancing axial compressive strength and increasing BMD, particularly in the dominant arm [25,26]. Muscle contraction seems to play a crucial role in bone microarchitecture alterations and seems to stimulate bone formation [27]. It is well reported that the dominant hand has better muscle strength and bone mass than the non-dominant side. This may be attributed to daily activities [28] and sports-specific demands [9,25,26] that maintain superior muscle function in the dominant upper limb.

**Figure 2:**
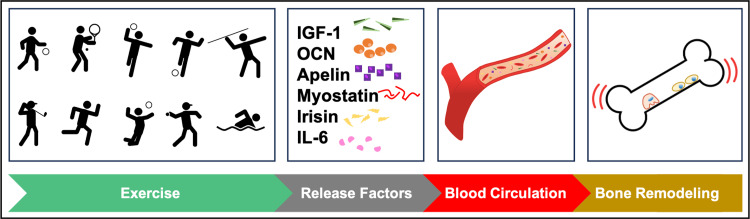
Exercise stimulates the release of important factors, such as insulin-like growth factor 1 (IGF-1), osteocalcin (OCN), apelin, myostatin, irisin, and interleukin 6 (IL-6), in the blood circulation and promotes bone remodeling Figure created by the authors

Healthy bones are sustained by continuous and balanced bone remodeling, which includes bone resorption and formation [29]. At the molecular level, exercise can promote bone strength by stimulating multiple factors, such as insulin-like growth factor 1 (IGF-1), osteocalcin (OCN), irisin, apelin, myostatin, and interleukin 6 (IL-6) (Figure 2) [30,31]. Particularly, the secretion of IGF-1 can regulate bone development and regeneration by acting directly and indirectly on target cells [30]. Exercise can also affect osteoblast function by the upregulation of OCN, a key determinant of bone formation [31]. In addition, apelin and myostatin, age-dependent myokines and positively associated with exercise, promote the osteogenic differentiation of human bone marrow mesenchymal stem cells (hBM-MSCs) to osteoblasts [30,31]. In the same direction, irisin has been shown to play a crucial role in osteoblast proliferation, differentiation, and mineralization, by increasing the level of osteogenic regulators, including Runt-related transcription factor 2 (Runx2), Osterix, alkaline phosphatase (ALP), and collagen 1 alpha 1 (Col1a1) [30,31]. Meanwhile, IL-6 is released during exercise and promotes osteoclast formation by stimulating the receptor activator of nuclear factor kappa-B ligand (RANKL) expression in osteoblasts via the IL-6 receptor, thus contributing to bone homeostasis [29,30].

To maximize bone health benefits, exercise should be dynamic rather than static [8]. Weight-bearing activities, such as soccer and endurance running, generate the necessary stress to stimulate new bone and are considered moderate to high in terms of bone-loading force [8]. In addition, muscle forces play a pivotal role in generating the mechanical stimulus in the bones during exercise, while jumping sports, such as volleyball and basketball, can provide a strong mechanical stimulus, in order to be positive and more beneficial to bone health, especially in the spine and the lower extremities [8,26]. In contrast, tennis and handball can transmit mechanical loads primarily in the upper extremities, especially the dominant arm, due to frequent muscle contractions [25,26,32].

Bone health in handball athletes

Team handball involves sudden torsional strains on the limbs and spine, which may significantly influence bone mass regulation [32]. The sport includes numerous jumps and sprints and frequently ball handling with the arms, resulting in considerable mechanical loading on both the upper and lower extremities. This indicates the osteogenic nature of handball [32-34]. Multiple studies have investigated the protentional positive role of handball training and participation on both BMD and BMC, underlining the higher BMD and BMC values in comparison to age-matched individuals with a physically inactive lifestyle [32,33,35-38] and active status with or without sport participation (Table 1) [9,32,34,35,38,39].

**Table 1:** Description of the included studies n, sample population; g, gender; F, female; M, male; BMD, bone mineral density; BMC, bone mineral content

Study	Participants (n/g)	Age (years)	Variables	Skeletal site	Comparisons	BMD and/or BMC results
Fagundes et al., 2022 [35]	115/F	15.5 ± 1.3	BMD (g/cm^2^) and BMC (g)	Upper limb, lower limb, trunk, ribs, and spine	Handball versus soccer and non-athletes	BMD values in handball players are higher for the trunk, ribs, and spine compared to soccer players (P < 0.05) and non-athletes (P < 0.001). BMC values in handball players are higher for the upper limbs, trunk, ribs, and spine compared to soccer players (P < 0.001) and for the lower limbs, trunk, ribs, pelvis, and spine compared to non-athletes (P < 0.001)
Hagman et al., 2021 [32]	35/F	63.9 ± 4.2	BMD (g/cm^2^) and BMC (g)	Proximal femur and lumbar spine	Handball versus football and untrained	BMD values in handball players are 8%-10% higher for the proximal femur and lumbar spine compared to elderly untrained control (P < 0.05). BMC values in handball players are 8%-13% higher for the legs and whole body compared to elderly untrained control (P < 0.001)
Pereira et al., 2021 [36]	41/F	68.3 ± 6.2	BMD (g/cm^2^) and BMC (g)	Lumbar spine and femoral neck	Handball versus inactive	BMD values for the lumbar spine and femoral neck are increased by 1.5% (P < 0.001) and 0.9% (P = 0.3), respectively, between baseline and after 16 weeks of handball training. BMC values for the lumbar spine and femoral neck are increased by 2.3% (P = 0.002) and 1.6% (P = 0.5), respectively, between baseline and after 16 weeks of handball training
Fristrup et al., 2020 [40]	14/M/14/F	24.1 ± 2.6	BMD (g/cm^2^) and BMC (g)	Leg and hip	Handball versus untrained	BMD values for handball players are higher for the hip (P < 0.001) compared to controls. BMC values for handball players are higher for the legs (P < 0.004) compared to controls
Krahenbuhl et al., 2018 [37]	29/F	14.4 ± 1.3	BMD (g/cm^2^) and BMC (g)	Lumbar spine, femoral neck, trunk, and arms	Handball versus inactive	BMD values in handball players are increased by 10.3% (P < 0.05) and 13.1% (P < 0.05) for the lumbar spine and femoral neck, respectively, compared to healthy adolescents. BMC values in handball players are increased by 12.1% (P < 0.05), 15.5% (P < 0.05), 11.9% (P < 0.05), and 10.4% (P < 0.05) for the lumbar spine, femoral neck, trunk, and arms, respectively, compared to healthy adolescents
Boshnjaku et al., 2016 [38]	30/F	22.3 ± 3.9	BMD (g/cm^2^)	Forearm (distal radius)	Handball versus soccer and untrained	BMD values for the dominant forearm are higher compared to the non-dominant forearm of handball players (P < 0.05) compared to the dominant forearm of control (P < 0.01) and soccer player (P < 0.05). BMD values for the non-dominant forearm are higher compared to the non-dominant forearm of control (P < 0.01) and soccer player (P < 0.05)
Missawi et al., 2016 [39]	50/M	10.8 ± 0.9	BMD (g/cm^2^) and BMC (g)	Lumbar spine, leg, hip, femoral neck, and arm (radius)	Handball versus active handball (dominant versus non-dominant side)	BMD values in handball players are higher for the lumbar spine, legs, and arm compared to non-active prepubescent boys. BMD values of the left side for the legs (P < 0.001) are higher compared to the right side in handball players. BMD values in handball players are higher for the right side for the arms compared to the left side in handball players. BMC values in handball players are higher for the lumbar spine, legs (P < 0.01), hip (P < 0.05), and arms compared to non-active prepubescent boys. BMC values of the right side for the arms (P < 0.05) are higher compared to the left side in handball players. BMC values of the right side for the hips are higher compared to the left side in handball players
Ubago-Guisado et al., 2015 [34]	20/F	9.8 ± 0.6	BMD (g/cm^2^) and BMC (g)	Pelvis, leg, hip, proximal femur, and arm	Handball (weight-bearing versus non-weight-bearing bones)	BMD values in handball players are higher for the legs (P < 0.05), trochanter (P < 0.05), and intertrochanter (P < 0.05) compared to swimming. BMD values in handball players are higher for the hip (P < 0.05) and intertrochanter (P < 0.05) compared to control. BMD values in handball players are higher for the arms compared to swimming, soccer, and controls. BMC values in handball players are higher for intertrochanter (P < 0.05) compared to basketball players. BMC values in handball players are higher for the hip (P < 0.05) and intertrochanter (P < 0.05) compared to control. BMC values in handball players are higher for the arms compared to soccer and controls
Bahri et al., 2013 [33]	17/F	15.8 ± 0.7	BMD (g/cm^2^) and BMC (g)	Lumbar spine, pelvis, leg, proximal femur, and arm	Handball versus inactive	BMD values in handball players are higher for the lumbar spine (P < 0.0001), femur (P < 0.001), legs (P < 0.015), and arms (P < 0.0001) compared to controls. BMC values in handball players are higher for the lumbar spine (P = 0.06), pelvis (P < 0.001), trochanter (P = 0.003), legs (P < 0.0001), and arms (P < 0.0001) compared to controls
Vicente-Rodriguez et al., 2004 [9]	24/F	14.2 ± 0.4	BMD (g/cm^2^) and BMC (g)	Lumbar spine, pelvis, leg, femur, and arm	Handball versus active	BMD values in handball players are higher for the pelvis (P < 0.05), legs (P < 0.05), and proximal femur (P < 0.05) compared to controls. BMD values in handball players are 3.3%, 6.4%, and 4.9% higher for the arms, spine and trochanter, respectively, compared to controls. BMC values in handball players are higher for the pelvis (P < 0.05), legs (P < 0.05), and spine (P < 0.05) compared to controls. BMC values in handball players are 5% and 7.3% higher for the arms and femoral neck, respectively, compared to controls

Although BMD naturally decreases with age and continues declining after 65 years in both genders [41], exercise appears to mitigate this decline, even in handball players [32,33,36]. Bahri et al. reported that the BMD of the lumbar spine (L1-L4) was 1.262 g/cm^2^ among teenage girls (15.8 years) practicing in handball [33]. Hagman et al. [32] found that handball training for approximately 43 years among female athletes at the age of 43.9 can reveal a BMD of 1.1 g/cm^2^, which seems high, but it is less when compared to younger handball players [36]. A total of 12% reduction can be observed among handball players over time, which seems to be less than 0.6%/year among older adults with a history of falls, underlying the importance of long-term exercise [41]. Interestingly, BMD values in the lumbar spine seem to be higher for handball players (0.969-1.262 g/cm^2^), when compared to age-matched soccer players (p < 0.05) and non-active individuals (p < 0.05) [32,35,36].

The BMD values of the lower limb, especially in the proximal femur (femoral neck and trochanter) of the handball players, were higher in comparison to active (p < 0.05) and non-active individuals (p < 0.05 to p < 0.001) [9,32,33,37]. Hagman et al. showed that BMD in female handball players was 9% higher for the femoral Ward’s triangle and 7% higher in the femoral trochanter than in age-matched untrained women [32]. Additionally, female adolescent handball players showed significantly higher BMD values (p < 0.05) compared to healthy adolescents who did not engage in regular physical activity or sports [37]. In this study, BMD increased by 13.1% and 13.2% for Ward’s triangle and the femoral neck, respectively. These results support the view that sport participation, such as handball, is an effective path to improving bone health during early years, potentially preventing bone diseases in older age.

**Figure 3:**
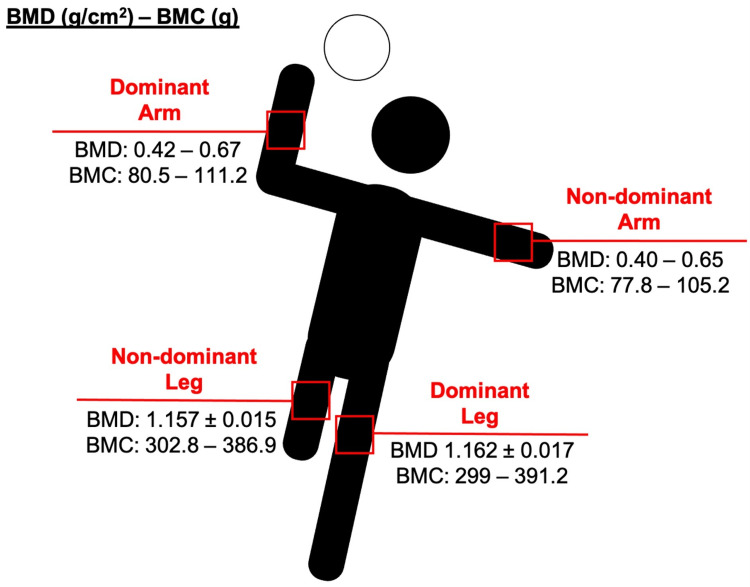
Side-to-side BMD/BMC comparisons among handball players Figure created by the authors BMD, bone mineral density; BMC, bone mineral content

On the other hand, it is interesting to note that handball induces mostly unilateral torsional strains in the upper extremities [34,39]. Boshnjaku et al. showed that BMD values for both dominant and non-dominant forearms were 19.6% (p < 0.01) and 15.7% (p < 0.01) higher, respectively, compared to untrained individuals and 12.9% (p < 0.05) and 5.3% (p < 0.05) higher, respectively, compared to soccer players [38]. Furthermore, the BMD values of the dominant forearm in handball players were significantly higher (p < 0.05) than those of the non-dominant forearm [38]. Vicente-Rodriguez et al. also found greater BMD in the dominant arm of handball players [9]. They noted that as little as three hours per week of handball participation led to significantly higher BMD values in the dominant arm compared to active counterparts. Similar results were reported by Missawi et al., who suggested that handball practice has a greater impact on the more stressed and dominant anatomical sites among prepubescent boys (Figure 3) [39]. This is understandable, as the dominant forearm of handball players functions as a weight-bearing site, being primarily responsible for dribbling and throwing the ball.

The literature contains limited data concerning bone health in male handball players. Missawi et al. demonstrated a beneficial effect in the most stressed anatomical sites among prepubescent boys who had engaged in at least two years of handball practice during the bone acquisition period [39]. Interestingly, they found increased BMD and BMC in the right dominant non-weight-bearing side due to ball contact and in the opposite weight-bearing side due to ground reaction forces. More recently, Fristrup et al. reported that handball training resulted in significant gains in the whole-body BMD and BMC for both male and female young adults [40]. Specifically, they found that handball training increased the regional leg BMC by 1.2% (p < 0.001) and hip BMD by 1.6% (p < 0.001). These findings further support the idea that intense interval exercise, such as handball, improves bone health, likely due to the high-impact strain stimulation imposed on the skeleton during training.

Data regarding the BMC of handball players and the effects of exercise align with the findings on BMD (Figure 3). Most studies have shown that the BMC of the trunk [35,37], pelvis [9,34], lumbar spine (L1-L4) [9,32,33,35,37,39], and ribs [35] in handball players is 8%-18% higher than in soccer players [35], swimmers [34], and both age-matched non-active [32-35,39] and active individuals [9,37]. Additionally, a 12-week handball training program with three supervised sessions per week led to a 1.2% increase in regional leg BMC (p < 0.05) [40]. This finding is consistent with Pereira et al., who reported a 2.2% increase in femur BMC (p = 0.004) following a 16-week recreational team handball-based exercise program [36]. The upper extremities are considered weight-bearing anatomical sites in handball athletes. Krahenbuhl et al. observed that handball players had significantly higher BMC (10.4%, p < 0.001) compared to a group of same-aged girls with no systematic physical activity [37]. Similar results were found when comparing young female soccer players to handball players. Fagundes et al. reported that adolescent women who had participated in handball training for at least one year presented higher BMC values than soccer players (p < 0.001) [35].

These findings collectively emphasize the osteogenic benefits of handball across various age groups, genders, and anatomical regions. When synthesizing the evidence, a clear pattern emerges: the sport’s dynamic, high-impact, and asymmetrical loading characteristics contribute to both site-specific and systemic improvements in BMD and BMC. Notably, the superior BMD and BMC values in handball players, especially in high-stress areas such as the lumbar spine, proximal femur, and dominant forearm, highlight how mechanical loading tailored through sports-specific movements can optimize skeletal adaptation [9,32,33,35,37-39].

Moreover, the early engagement in handball during critical growth periods, such as prepubescence and adolescence, appears to yield long-term skeletal benefits, potentially offsetting the age-related decline in bone mass seen later in life [33,36,39,41]. Interestingly, while the forearm benefits from repetitive unilateral impact, the femoral and spinal regions benefit from the ground reaction forces generated during sprinting, pivoting, and jumping [9,32,34,38,40]. This dual-modality loading, comprising both axial and torsional strains, may explain the broader and more pronounced skeletal improvements seen in handball players compared to athletes in less mechanically demanding sports such as swimming or even soccer [34,35]. Taken together, these correlations reinforce the notion that targeted, sports-specific loading regimens can serve as powerful tools for enhancing bone health and mitigating osteoporosis risk, especially when introduced during developmental years and sustained throughout adulthood [9,32-36,39-41].

Limitations

Although the available evidence consistently indicates that handball participation enhances BMD and BMC, several limitations should be noted. Most studies are cross-sectional, preventing causal conclusions about training and bone adaptations. Reported BMD gains vary across age groups, likely reflecting differences in bone remodeling rates and training duration, with adolescents generally showing greater improvements than adults. Dominant-to-non-dominant limb asymmetries, while reflecting adaptive bone responses, may pose clinical concerns such as uneven loading or injury risk. Additional limitations include variability in DEXA calibration across studies, a predominance of female participants, and limited longitudinal follow-up beyond adolescence, which restricts the understanding of long-term effects on bone health. Reporting effect sizes or average percentage increases in BMD and BMC would strengthen future analyses.

## Conclusions

Handball players demonstrate higher BMD and BMC values compared to their age-matched non-active and active peers, as well as athletes practicing in low-impact sports, such as swimming. Handball participation appears to produce superior BMD/BMC values on the dominant side, due to the increased mechanical stress on the dominant arm and leg compared to the contralateral side. Despite the side-to-side asymmetries in BMD/BMC values, handball effectively stimulates osteogenic responses in both upper and lower extremities. As a result, handball participation appears to be an effective osteogenic exercise modality and may contribute to bone health across the lifespan.
